# Relationship between students’ perceived parent–teacher–student relationships, sense of school belonging, and well-being in China: investigating the difference between primary and secondary students

**DOI:** 10.3389/fpsyg.2026.1906317

**Published:** 2026-09-21

**Authors:** Bingwen Lou, Xingyue Yang

**Affiliations:** 1 School of Law, Southeast University, Nanjing, China; 2 Longchang No. 7 Middle School, Neijiang, China

**Keywords:** student well-being, sense of school belonging, parent–teacher–student relationships, mediating effect, primary and secondary school students

## Abstract

**Objectives:**

Students’ well-being is a critical indicator of their academic performance, pro-social behavior, and future development. Students’ perceived relationships with parents, teachers, and peers, along with their sense of school belonging, are significantly associated with their well-being. This study aimed to examine whether students’ sense of school belonging mediates the relationship between perceived parent–teacher–student relationship and well-being. The study also explored whether there were differences between primary and secondary school students on the main dimensions.

**Methods:**

Data were obtained from the Organisation for Economic Co-operation and Development Survey on Social and Emotional Skills, which included responses from 6,742 students in Suzhou, China (3,508 Grade 4 and 3,234 Grade 10 students). A *t*-test was used to determine whether any differences existed in the five dimensions between students in Grades 4 and 10. Correlation and regression analyses were used to analyze the relationship between different variables. Structural equation modeling with a bootstrap resampling procedure was employed to test the mediation model.

**Results:**

The findings showed that primary school students reported positive relationships with parents and peers, greater overall well-being, and a stronger sense of school belonging than secondary school students. However, they reported less positive relationships with their teachers. After controlling for other variables, the findings indicated that students’ perceived parent–teacher–student relationships and sense of school belonging were positively and significantly related with students’ well-being. These four dimensions collectively explain 44.8% of the variation in the differences in students’ sense of well-being. The results of the bootstrap mediation analysis showed that students’ sense of school belonging significantly partially mediated the relationship between perceived parent-teacher-student relationships and well-being.

**Conclusion:**

Well-being serves as an important indicator that is positively associated with students’ current learning and development and may be relevant to their future success. This study provides empirical evidence that a sense of school belonging serves as a key mediating mechanism through which perceived parent-teacher-student relationships translate into students’ well-being, extending the application of belongingness theory and self-determination theory within the Chinese educational context. It is imperative for school authorities, teachers, and parents to create a learning environment that promotes students’ aspirations, well-being, and academic development.

## 1 Introduction

The need to belong is a fundamental and pervasive psychological motivation, and human beings are innately driven to form and maintain lasting, positive, and meaningful interpersonal relationships (Baumeister and Leary, 1995). Within the school context, satisfaction of the need to belong contributes to a sense of school belonging, which is described as “the extent to which students feel personally accepted, respected, included, and supported by others in the school social environment” (Goodenow and Grady, 1993). When students’ need to belong is satisfied, they show greater interest in learning activities (Encina and Berger, 2021), have lower dropout rates (McWhirter et al., 2018), and engage in fewer internalizing and externalizing behaviors (Arslan, 2022; Parr et al., 2020; Kidger et al., 2012). A growing body of research suggests that students’ sense of school belonging, a key indicator of their well-being and performance, has been declining since the Programme for International Student Assessment (PISA) 2003 (OECD, 2017). Meanwhile, a study involving Swedish students showed that the decline in school belonging was most pronounced among low-achieving, working-class, and foreign-born students (Högberg and Lindgren, 2023). In the wake of rapid social development in China, students’ well-being has garnered increasing attention from policymakers and researchers. A meta-analysis of 1,043 articles on students’ mental health between 2010 and 2020 showed that mental health issues such as anxiety, depression, and suicide attempts have become increasingly common among students (Yu and Huang, 2023). Accordingly, it is imperative to focus on students’ well-being so that schools, parents, and teachers can create an environment that promotes students’ well-being.

Bronfenbrenner (1979) proposed that the environment can be regarded as “a set of layered nested structural systems” and emphasized that individual development depends on the continuous and two-way interaction between people and the surrounding multiple ecosystems. The framework is examined in the school field. Parents, teachers and peers constitute the most influential relationship nodes in the microsystem. Students not only frequently contact these objects in daily communication, but also often obtain emotional confirmation, practical support and behavioral guidance from them. Furthermore, if this ecological perspective and the theory of belonging needs are examined by juxtaposition, the two have obvious complementary characteristics in the interpretation mechanism: the former clarifies the key interpersonal sources relied on to meet the needs of belonging, while the latter reveals what kind of psychological intermediary path the above-mentioned interpersonal interaction uses to eventually transform into students’ inner connection experience and perception of acceptance. The need-to-belong theory points out that satisfying the need for belonging requires individuals to maintain frequent and emotionally positive interactions with a few others. These interactions also need to be embedded in a relationship framework that is temporally continuous and stable and involves mutual concern for each other’s well-being (Baumeister and Leary, 1995). In the school setting, the relationships between students and their parents, teachers, and peers can usually meet the above requirements, because these relationships often provide repeated positive interactions within a relatively stable social structure. A large number of studies have confirmed that these three relational sources are important predictors of students’ sense of school belonging (Allen et al., 2018; Dukynaite and Dudaite, 2017), and studies also show that the effect of emotional support from parents and peers is particularly prominent (Adinata et al., 2025; Longaretti, 2020). From this, it can be inferred that the quality of students’ perceived parent-teacher-peer relationships may be related to the extent to which their belonging needs are satisfied, and this satisfaction level may affect the formation of their sense of school belonging.

The need for belonging is a basic human psychological need, but the extent to which it is realized is affected by the specific situation and constrained by the two mechanisms of saturation and substitution (Baumeister and Leary, 1995). When existing relationships are sufficient to meet this demand, the individual’s intention to seek new relationships declines; and relationship resources from different sources can also substitute for each other within a certain range. The relative importance of these mechanisms may vary at different stages of development. For primary school students, the quality of the teacher-student relationship is the strongest factor in predicting students’ well-being (Valcke et al., 2022). However, in the upper grades of primary school, students’ sense of closeness to teachers tends to decline from fifth grade to sixth grade (Furrer and Skinner, 2003). After entering secondary school, social relations change dramatically, and peer relationships become increasingly prominent, as teenagers begin to pursue more autonomy and gradually distance themselves from their families (Eccles, 1999). Erikson’s theory of psychosocial development (Erikson, 1968) helps to clarify this developmental trajectory: the construction of self-identity enhances the need for peer recognition and autonomy, thus shifting the focus from adults to peers. This developmental change may mean that the threshold for satisfying the need for belonging in adolescence rise, requiring more frequent and higher-quality interaction to achieve the same level of satisfaction. At the same time, the substitutability between different sources of relationships may weaken; for example, even if teachers provide support, it may be increasingly difficult to compensate for the shortcomings caused by poor peer relationships. These changes may make teenagers more susceptible to social exclusion, and studies have shown that social exclusion can directly impair their well-being and mental health (Arslan, 2018a, 2018b). This study intends to compare students in primary and secondary schools, because according to the developmental framework, the contribution of various relationship resources to school belonging and well-being may differ at different stages.

Previous studies have focused on the parent–child relationship (Ge, 2020; Chidambaram et al., 2023), the teacher-student relationship (Cornelius-White, 2007; Harding et al., 2019; Zhou, 2023), and peer relationships (Nie et al., 2022; Kokkonen et al., 2021), which have verified their predictive roles on students’ well-being. At the same time, the formation of a sense of belonging in schools has also been confirmed to be significantly influenced by parental involvement (Ahmadi and Ahmadi, 2020; Kuperminc et al., 2008), teacher support (Chiu et al., 2016; Allen et al., 2016), and peer acceptance (Osterman, 2000; Thijs et al., 2019). Furthermore, school belonging has been established as a mediating mechanism between the above-mentioned interpersonal resources and multiple developmental outcomes, which cover academic performance (Ahmadi et al., 2020; Arslan, 2022; Liu et al., 2022), internalizing and externalizing problem behaviors (Arslan, 2022), and school adjustment (Ibrahim and El Zaatari, 2019). Despite the accumulation of the above evidence, there are still obvious fragmented characteristics in the current understanding. Few studies have attempted to integrate the three types of relational variables within the same analytical framework and systematically evaluate their joint effects on well-being through school belonging.

Studies have confirmed the significant effects of parent–child, teacher-student, and peer relationship each affect students’ school belonging and well-being. Many studies have focused on a single educational stage, such as vocational high school students (Adinata et al., 2025), secondary school students (Saxer et al., 2025; Kuperminc et al., 2008), or the primary-secondary transition (Longaretti, 2020). Cross-stage comparisons remain rare, even though developmental theories predict that the relative importance of these relationships shifts across educational stages (Eccles, 1999). In the Chinese context, research has examined school belonging among vocational high school students (Adinata et al., 2025), but whether this mediating mechanism differs across primary and secondary stages remains unclear. The present study addresses this gap by testing school belonging as a mediator linking parent-teacher-student relationships to well-being and comparing primary with secondary students.

## 2 Theoretical perspectives and hypotheses

### 2.1 Students’ perceived parent–teacher–friend relationships and well-being

Bronfenbrenner’s (1979) ecological systems theory emphasizes the multi-layered nested structure of the environment, and individual development arises from two-way interaction with various ecological levels. In schools, parents, teachers, and peers form the core relational agents of the microsystem, which are crucial to students’ daily interactions and support-seeking. The need-to-belong theory (Baumeister and Leary, 1995) explains how this kind of relationship affects well-being: the need to belong, as a fundamental and universal motivation, requires individuals to maintain frequent and non-aversive interactions in lasting relationships. When students perceive care and acceptance from the three parties, the need to belong is satisfied, which is conducive to positive emotions and physical and mental health; on the contrary, long-term frustration can easily cause anxiety, depression, and other psychological distresses, which undermine overall well-being. It can be seen that ecological systems theory points out the relational sources, and the need-to-belong theory clarifies the path of action: the perceived quality of interactions determines whether the need to belong is satisfied, which in turn affects affective outcomes. The combination of the two theories links the parent–teacher–peer relationship perceived by students with their well-being.

First, the parent–child relationship is of great significance because it constitutes an important link between the family microsystem and the school microsystem. The emotional support, meticulous care and active intervention provided by parents all reflect the unique, lasting, and stable caring characteristics of the family microsystem. When students can perceive this support, their need for belonging can be met, which helps to maintain emotional health and overall psychological well-being. Cross-cultural research has shown that parental support is one of the most significant variables predicting the well-being of adolescents (Wu and Lee, 2022). Similarly, Fino et al. (2025) found that the parental support perceived by early adolescents was significantly positively correlated with their emotional well-being. Studies have shown that parental involvement and their relationship with their children play mediating roles in children’s well-being (Ge, 2020). Low levels of well-being among children may be associated with a range of factors, including living in a single-parent household, strained child–parent relationship, and family conflicts (Kokkonen et al., 2021; Patalay and Fitzsimons, 2016), Similarly, parental neglect was also been found to be negatively associated with emotional well-being (Chidambaram et al., 2023).

Second, the teacher–student relationship, as a core element of the school microsystem, serves as another important source of students’ need to belong. In the teacher–student relationship, daily classroom interactions provide frequent, non-aversive interactions. Positive teacher–student relationships are characterized by teachers’ care, respect, dedication, and support for students, thereby creating a learning community marked by respect, equality, harmony, and camaraderie. They promote students’ well-being and mitigate risky behaviors (Cornelius-White, 2007; Jamal et al., 2013; OECD, 2017). Studies have also explored the association between teacher and student well-being, demonstrating a positive relationship between teacher well-being, teacher–student relationship, and student well-being (Harding et al., 2019). In the school contexts in Germany, England and Wales, researchers linked teacher data with student data and found that the higher the teachers’ own level of well-being, the higher the students’ well-being, and at the same time, the frequency of students’ psychological distress and problem behaviors decreased (Bilz et al., 2022; Harding et al., 2019). In addition, a study of 748 Chinese college students also showed that there was a significant positive correlation between the positive quality of the teacher-student relationship and the subjective well-being of students (Zhou, 2023). Conflicts between teachers and students are identified as important risk factors that undermine students’ well-being (Saxer et al., 2025).

Finally, peer relationships, also situated within the school microsystem, serve as a third critical source for students’ need to belong. Peer friendships and acceptance provide students the frequent, non-aversive interactions needed to establish the lasting, positive connections that, according to need-to-belong theory, are essential for satisfying this fundamental need. Studies have shown that peer support is significantly associated with subjective well-being among both early adolescents (Fino et al., 2025) and middle school students (Wang et al., 2025). Many studies have demonstrated that negative peer relationships have detrimental effects on students’ well-being. Bullying, mainly physical and cyberbullying, is considered the most distressing threat to student well-being (Nie et al., 2022; OECD, 2017). Data from 114 adolescents also indicated that discrimination by peers was associated with significantly lower mental well-being (Kokkonen et al., 2021), with formation and maintenance of positive interpersonal relationships playing a vital role in promoting healthy development and overall well-being (Arslan, 2018c). Overall, the three relational sources are not simply additive, but complement each other. The emotional security given by parents, the recognition expressed by teachers, and the acceptance provided by peers jointly shape a unified sense of social worth, that is, individuals perceive themselves as recognized and valued by the school community.

Thus, parent–teacher–student relationships function as a key interpersonal resource that may be associated with student well-being, depending on the quality of care and support perceived. Accordingly, the following hypothesis is proposed:

*H1:* Parent–teacher–student relationships are positively associated with student well-being.

### 2.2 The mediating role of sense of school belonging

Need-to-belong theory suggests that the need to belong is a fundamental human motivation (Baumeister and Leary, 1995), which drive individuals to establish and maintain stable and positive interpersonal relationships. In terms of the school situation, the satisfaction of this need is mainly reflected in the students’ sense of belonging to the school, which is defined as the degree to which students perceive acceptance, respect, inclusion, and support from others in the school social environment (Goodenow and Grady, 1993; Arslan and Duru, 2017). This sense of belonging is not only the internalized product of the satisfaction of belonging needs in the school context, but also the key mediating mechanism in the transformation of interpersonal experience into individual well-being. In this transformation process, parents, teachers and peers all play a role in their own unique ways. According to Bronfenbrenner’s (1979) ecological systems theory, in the interactive interface between family and school, that is, the mesosystem, parental participation constitutes an important dimension of the school environment and directly plays a role in the process of students’ cognitive formation and experience of school belonging. Students spend a substantial amount of their time in school, underscoring the importance of making them feel part of their educational environment (Encina and Berger, 2021). Many studies have shown that students with a high sense of school belonging not only show a more positive attitude towards academic tasks (Encina and Berger, 2021), but also significantly reduce the risk of dropping out (McWhirter et al., 2018). In addition, students tend to have a lower frequency of internalizing and externalizing problems (Arslan, 2021, 2022; Parr et al., 2020). At the same time, there is a positive relationship between the sense of school belonging and academic achievement. A strong sense of belonging can often enhance academic performance (Arslan, 2022; Pittman and Richmond, 2008; Sánchez et al., 2005; Smith et al., 2021). Therefore, cultivating and enhancing students’ sense of school belonging should be regarded as an important issue in educational practice, because schools play an irreplaceable role in the growth of students (Korpershoek et al., 2019).

According to the need-to-belong theory, the sense of school belonging does not come solely from passive interpersonal interaction, but can also be understood as a psychological characteristic that gradually emerges after individuals feel that the need for belonging is met. When students feel acceptance from people around them, this perception is often internalized as identification with the school community, and acts as a mediator between interpersonal experiences and individual well-being. Studies have pointed out that parents, teachers and peers play an indispensable role in the process of cultivating students’ sense of school belonging (Dukynaite and Dudaite, 2017). Although these three relational sources contribute to school belonging, the unique role of the student-parent relationship cannot be ignored. Indeed, some scholars believe that the impact of family variables on the academic performance and behavior of adolescents may even exceed the impact of school-level factors (Duncan et al., 2001). Parents have always been at the heart of their children’s growth. For example, family income and parents’ education level have been found to be significantly related to students’ sense of school belonging and academic ability (Hernández et al., 2014). In addition, parental participation not only directly affects students’ sense of school belonging, but also may indirectly promote their academic adaptation and life satisfaction through this psychological mechanism (Ahmadi and Ahmadi, 2020; Kuperminc et al., 2008). Family cultural values should not be overlooked. Studies show that stronger familism is often associated with fewer depressive symptoms and a higher sense of school belonging (Cupito et al., 2015).

In recent years, the positive correlation between the teacher-student relationship and the sense of school belonging has been supported by empirical research (Chiu et al., 2016). On this basis, subsequent studies have further shown that teacher support can indirectly have a positive impact on students’ academic performance and life satisfaction by strengthening this sense of school belonging (Ahmadi and Ahmadi, 2020; Chang and Bangsri, 2020). In fact, students’ perceived school belonging is shaped by the quality of the teacher-student interactions and peer support (Allen et al., 2016; Roorda et al., 2011). When students are under academic pressure, a good teacher-student relationship often acts as a protective resource to help prevent or alleviate learned helplessness (Raufelder and Kulakow, 2021). If students perceive that teachers are fair (Klem and Connell, 2004; Wong et al., 2022), caring, respectful, and trustworthy, they are more likely to have a strong sense of school belonging (Chang and Bangsri, 2020; Ibrahim and El Zaatari, 2019). For students who suffer from bullying on campus, teacher support also paly a crucial role in fostering their sense of school belonging (Arslan, 2021, 2022; Parr et al., 2020). In recent years, the positive correlation between the teacher-student relationship and the sense of school belonging has been supported by empirical research (Chiu et al., 2016). On this basis, follow-up research further found that teacher support can indirectly have a positive impact on students’ academic performance and life satisfaction by improving their sense of school belonging (Ahmadi and Ahmadi, 2020; Chang and Bangsri, 2020). In fact, students’ perception of school belonging depends on the quality of the teacher-student relationship and peer support, which together form the basis of this perception (Allen et al., 2016; Roorda et al., 2011). When students are under academic pressure, a good teacher-student relationship often acts as a protective resource to help prevent or alleviate learned helplessness (Raufelder and Kulakow, 2021). If students perceive that teachers are fair (Klem and Connell, 2004; Wong et al., 2022), caring, respectful, and trustworthy, they are more likely to have a strong sense of school belonging (Chang and Bangsri, 2020; Ibrahim and El Zaatari, 2019). For students who suffer from bullying on campus, teacher support is important for cultivating their sense of school belonging (Arslan, 2021, 2022; Parr et al., 2020), and the effect of this support has been confirmed to be quite prominent in existing studies (Allen et al., 2016).

Peer relationships also play a key role in cultivating students’ sense of school belonging (Dehuff, 2013). If students have friends at the school or are part of a close peer circle, they can often feel respect and recognition from others, and thus form a deeper sense of school belonging (Delgado et al., 2015). Friendships on campus can not only enhance teenagers’ sense of school belonging and educational well-being (Hamm and Faircloth, 2005; Vaquera, 2009), but also have a significant impact on their behavior, values, and attitudes (Berndt, 1999). Good peer interaction helps to promote classroom identity, which is one of the basic components of students’ sense of school belonging (Thijs et al., 2019). In addition, previous studies have emphasized that the role of the degree of student acceptance in shaping the sense of school belonging cannot be ignored (Osterman, 2000; Urga, 2008).

There is a positive relationship between a sense of school belonging and students’ well-being, and well-being helps to form positive self-identification in adolescence (Brechwald and Prinstein, 2011). Students who have a strong sense of school belonging are often more willing to behave prosocially (Arslan et al., 2020), and their life satisfaction is also higher (Ahmadi and Ahmadi, 2020), thus promoting overall well-being (Arslan et al., 2020). According to the need-to-belong theory, when this need is fully met, individuals are prone to positive emotions and achieve good psychological development; on the contrary, once the need to belong is hindered, it may cause psychological distress and difficulties in adaptation (Baumeister and Leary, 1995). From this point of view, the sense of school belonging, as a key indicator of whether an individual’s need to belong is satisfied in the environment, acts as a mediator between interpersonal experiences and well-being. Data from 1,087 migrant children in China showed that students’ sense of school belonging mediated the effect of bullying on mental health (Nie et al., 2022). The findings of studies based on other school cultural contexts align with this conclusion (Aldridge and McChesney, 2018; Kokkonen et al., 2021).

Thus, this sense of belonging may serve as a mediating mechanism through which interpersonal relationships are associated with student well-being. Accordingly, the following hypothesis is proposed:

*H2:* Sense of school belonging mediates the relationship between parent–teacher–student relationships and student well-being.

### 2.3 Differences between primary and secondary students

The need-to-belong theory points out that belonging is a fundamental human need, but whether it can be satisfied often depends on the specific environment. This process occurs through two mechanisms: saturation and substitution (Baumeister and Leary, 1995). When an individual’s existing relationships are enough to meet this need for belonging, the motivation to establish new relationships will be weakened; similarly, different types of interpersonal relationships can also substitute for one another to a certain extent. The relative prominence of these mechanisms may vary across developmental stages. In primary school, the quality of teacher-student relationships is the most predictive of school well-being (Valcke et al., 2022), but students’ sense of closeness to teachers gradually weakens from the fifth grade to the sixth grade (Furrer and Skinner, 2003). After entering middle school, social relations undergo significant adjustment, and peer relationships become increasingly important, which is in line with the trend of teenagers pursuing autonomy and alienating themselves from families (Eccles, 1999). This developmental shift may imply that the threshold for satisfying belonging needs rises in adolescence, and meeting requires more frequent and higher-quality interactions to achieve comparable levels of fulfillment. At the same time, the substitutability between different sources of relationships may decline, for example, the compensatory effect of teacher support in dealing with peer difficulties may be weakened. These changes make adolescents more susceptible to social exclusion, which has been proven to directly impair their mental health and life satisfaction (Arslan, 2018a, 2018b). However, the substitution mechanism is still of practical significance at this stage. The social circle of secondary students is more diverse, covering out-of-school friends, online communities and interest groups (Cai et al., 2023), which can provide substitutive support when school belonging is insufficient. However, such external resources may also dilute the additional contribution of school belonging to overall well-being, thus weakening its explanatory power among secondary students.

This developmental process is inherently consistent with Erikson’s (1968) theory of psychosocial stages. According to the theory, the core challenge faced by individuals in adolescence is to form a stable identity, which intensifies their desire for peer acceptance and autonomy, thereby changing the weight of adult-peer relationships in the individual’s social network. Previous research findings have also shown that secondary students’ sense of school belonging is usually lower than that of primary students (Morinaj and Hascher, 2022; Neel and Fuligni, 2012; Johnson, 2008). The reasons for this difference are more complicated. Adolescents often find it difficult to adapt to the school climate and organizational structure of secondary schools (Johnson, 2008); the institutional practices sometimes fail to adequately address students’ psychological needs for belonging (Goodenow and Grady, 1993). These issues precisely reflect the real dilemma of the decline in the substitutability of relationship sources mentioned earlier. Similarly, secondary students’ overall well-being tends to be lower than that of primary students. For example, in a cross-grade comparision of well-being, Tobia et al. (2019) found that the scores of secondary girls were significantly lower than those of primary school counterparts. In addition, a large-sample survey of 8,285 Finnish students in grades 4 to 12 also showed that the level of well-being of junior students was higher than that of senior students within the same school (Konu and Lintonen, 2006). This trend of declining in well-being aligns with theoretical expectations. When the threshold of students’ demand for a sense of school belonging rises, it is difficult for the interpersonal support in the school setting to fully meet this need. At the same time, the buffering effect of the sense of school belonging on the psychological well-being of secondary students is also weakening.

Thus, school level may moderate the process through which interpersonal relationships influence student well-being, with school belonging serving as a mediator. Accordingly, the following hypothesis is proposed:

*H3:* School level (primary vs. secondary) moderates the relationship between sense of school belonging and student well-being, such that the positive association is stronger for primary school students than for secondary school students.

## 3 Methods

### 3.1 Sample

This study used data from the Organisation for Economic Co-operation and Development (OECD) Survey on Social and Emotional Skills (SSES), which not only examines the factors related to the formation and growth of students’ social and emotional skills, but also delves into aspects such as students’ well-being and sense of school belonging. The SEES employed a stratified two-stage cluster sampling design in Suzhou (Jiangsu Province, China). Schools were stratified by district and school type, and a total of 151 schools were selected, and 50 students per school were sampled, resulting in a final sample of 7,246 students aged 10 and 15 years who participated in the survey in 2019. The study excluded students other than Grades 4 and 10, and samples with missing values on key variables were removed by listwise deletion. The final analytical sample consisted of 6,742 students, of whom 3,508 were from Grade 4 (52.0%) and 3,234 from Grade 10 (48.0%). This study was conducted as a secondary data analysis of a publicly available dataset. The original data collection in Suzhou was carried out in accordance with both OECD ethical protocols and local institutional requirements, with informed consent obtained from participants’ legal guardians. As the present study involved only the analysis of de-identified, publicly accessible secondary data, no additional institutional review board approval was required.

### 3.2 Measures

The measures included perceived relationship with parents, teachers, and students; sense of school belonging questionnaire, WHO Well-Being Index, and student grade.

#### 3.2.1 Parent–teacher–student relationship questionnaire

The parent–teacher–student relationship questionnaire had three subdimensions. The first was perceived relationship with parents (*RELPARENTS*), in which students were asked to respond to the following items: *“I get upset easily with my parents,” “It is difficult to talk with my parents*,*”* and *“I feel angry at my parents*.*”* Four statements were provided: “almost never or never true.” “sometimes true,” “often true,” and “almost always or always true.” The items were revised prior to the analysis given that they were negatively worded.

The second dimension concerned perceived relationship with teachers (*RELTEACH*). Students were asked to indicate how often they experienced the following in the past 12 months: *“Most of my teachers treated me fairly,” “I got along well with most of my teachers,”* and *“Most of my teachers were interested in my well-being*.*”* Students were given the following response options: “never or almost never,” “a few times a year,” “a few times a month,” or “once a week or more.”

The third dimension represented perceived relationship with friends (*RELFRIENDS*). It included four items: *“My friends understand me,” “My friends accept me as I am,” “My friends are easy to talk to”* and *“My friends respect my feelings*.*”* Four statements were given: “almost never or never true,” “sometimes true,” “often true,” and “almost always or always true.”

#### 3.2.2 School belonging questionnaire

This questionnaire was adapted from the School Belong Scale in the student questionnaire for the Programme for International Student Assessment (PISA) (OECD, 2003). The original questionnaire included six items, comprising three positive and three negative statements. The positive statement items that indicated students’ school belonging were: *“I make friends easily at school,” “I feel like I belong at school,”* and *“other students seem to like me.”* Students were given the following response options: 1 for “strongly disagree,” 2 for “disagree,” 3 for “agree,” and 4 for “strongly agree.”

#### 3.2.3 WHO-5 well-being index

The 5-item WHO Well-Being Index (WHO-5) is one of the most widely used questionnaires to assess subjective psychological well-being; it has been translated into more than 30 languages and has been used in different research projects globally (Topp et al., 2015). Five statements related to well-being were included in this (*WELLBEING*) index. Students were asked whether they experienced the following symptoms over the past 2 weeks by selecting one of five options (“at no time,” “some of the time,” “more than half of the time,” “most of the time” or “all the time”). The five items were described as *“Felt cheerful and in good spirits,” “Felt calm and relaxed,” “Felt active and vigorous.” “Woke up feeling fresh and rested,”* and *“Daily life filled with things that interest me*.*”*

### 3.3 Data analysis

SPSS22.0 and Mplus 8.4 packages were employed to analyze the data. First, exploratory factor analysis was conducted to determine whether there was common method bias. The results indicated that none of the five dimensions accounted for more than 40% variance, suggesting the absence of common method bias (Podsakoff et al., 2003).

Second, confirmatory factor analysis was performed to evaluate each measurement model fit. It was found that the three dimensions had a good fit with root mean square error of approximation (RMSEA) (0.040) < 0.08, comparative fit index (CFI) (0.990) > 0.9, Tucker–Lewis index (TLI) (0.986) > 0.9, and standardized root mean square residual (SRMR) (0.027) < 0.08 (Hu and Bentler, 1999). Since *χ*^2^ is affected by the sample size, *χ*^2^/*df* was abandoned as a measure of model fit in subsequent reports (Tay and Drasgow, 2012). The reliability and validity of the parent–teacher–student relationship were 0.793 and 0.806, respectively, and the factor loading of the 10 items was greater than 0.6. As there are only three items in the students’ school belonging dimension, the confirmatory factor analysis can be considered a saturation model. Reliability and validity were 0.720 and 0.674, respectively, and factor loading for the three items ranged from 0.616 to 0.746. The WHO-5 Well-Being Index also had a good fit with RMSEA (0.073) < 0.08, CFI (0.990) > 0.9, TLI (0.980) > 0.9, and SRMR (0.014) < 0.08. Reliability and validity were 0.891 and 0.883, respectively, and factor loading for the five items ranged from 0.775 to 0.820.

Third, various methods were employed to test the research hypothesis. A t-test was used to determine whether any differences existed in the five dimensions between students in Grades 4 and 10. Correlation and regression analyses were used to analyze the relationship between different variables. Three structural equation models were constructed to explore the direct and indirect influences of students’ perceived parent–teacher–student relationship and sense of school belonging on well-being.

## 4 Results

### 4.1 Descriptive statistics and correlation of variables

Table 1 presents the mean, standard deviation, and correlation among the five dimensions. As all questionnaires employed a 4-point Likert scale, the mean for all explanatory variables remained above 3.1, suggesting that students in Suzhou generally perceived positive relationships with parents, teachers, and peers, and reported overall high levels of well-being. The mean score of the students’ sense of school belonging was 2.94, below the threshold. As expected, the four predicted factors were positively related to well-being (all *ps* < 0.001), and the value of *r* varied between 0.14 and 0.50. Moreover, students’ well-being was positively correlated with their sense of school belonging (*r* = 0.50, *p* < 0.001), perceived relationships with parents (*r* = 0.40, *p* < 0.001), teachers (*r* = 0.24, *p* < 0.001), and peers (*r =* 0.39, *p* < 0.001).

**Table 1:** Descriptive statistics of variables for students in Grades 4 and 10.

Variables	*M*	SD	1	2	3	4
1. Perceived relationship with parents	3.36	0.63				
2. Perceived relationship with teachers	3.33	0.78	0.14^***^			
3. Perceived relationship with friends	3.12	0.69	0.19^***^	0.27^***^		
5. Sense of school belonging	2.94	0.59	0.27^***^	0.32^***^	0.49^***^	
4. WHO-5 Well-Being Index	3.30	0.93	0.40^***^	0.24^***^	0.39^***^	0.50^***^

^***^p < 0.001.

M, mean; SD, Standard Deviation.

The differences between Grades 4 and 10 students across the five dimensions were examined. The t-test results showed that, on average, compared to Grade 10 students, Grade 4 students perceived positive relationships with their parents (*M*_Grade 4_ = 3.55, *M*_Grade 10_ = 3.15, *t* (1, 6,740) = 28.01, *p* < 0.001) and peers (*M*_Grade 4_ = 3.20, *M*_Grade 10_ = 3.02, *t* (1, 6,740) = 11.19, *p* < 0.001), and negative relationships with teachers (M_Grade 4_ = 3.24, M_Grade 10_ = 3.43, *t* (1, 6,740) = −10.63, *p* < 0.001). On average, Grade 4 students reported a higher sense of school belonging (*M*_Grade 4_ = 3.05, *M*_Grade 10_ = 2.83, *t* (1, 6,740) = 15.52, *p* < 0.001) and greater overall well-being (*M*_Grade 4_ = 3.59, *M*_Grade 10_ = 2.98, *t* (1, 6,740) = 25.58, *p* < 0.001) compared to their counterparts in Grade 10.

### 4.2 The direct effect of parent–teacher–friend relationships on well-being

Three regression models were constructed to explore the effect of perceived parent–teacher–friend relationships on students’ well-being. The first, which included all students, showed a good fit with RMSEA (0.040) < 0.08, CFI (0.983) > 0.9, TLI (0.979) > 0.9, and SRMR (0.034) < 0.08. For the Grade 4 sample, the regression model had a good fit, with RMSEA (0.032) < 0.08, CFI (0.989) > 0.9, TLI (0.986) > 0.9, and SRMR (0.021) < 0.08. For Grade 10 students, the regression model had a good fit, with RMSEA (0.046) < 0.08, CFI (0.978) > 0.9, TLI (0.973) > 0.9, and SRMR (0.038) < 0.08. After controlling for other variables, students’ perceived relationships with parents, teachers, and peers explained 32.9% of variance in well-being in the full sample, 32.6% in the Grade 4 sample, and 24.0% in the Grade 10 sample. Table 2 presents the unstandardized coefficients. First, students’ perceived relationship with their parents positively predicted well-being across all three models (*p* < 0.001). The unstandardized coefficients were 0.523 for the full sample, 0.407 for Grade 4, and 0.436 for Grade 10. Second, students’ perceived relationship with teachers was also a significant positive predictor in all models (*p* < 0.001). The unstandardized coefficients were 0.142 for the full sample, 0.259 for Grade 4, and 0.191 for Grade 10. Third, students’ perceived relationship with friends was consistently and positively associated with well-being across all models (*p* < 0.001). The respective coefficients were 0.389 for the full sample, 0.346 for Grade 4, and 0.376 for Grade 10.

**Table 2:** Estimate of regression models.

Predict variables	Model 1 (Full sample)		Model 2 (Grade 4 sample)		Model 3 (Grade 10 sample)	
	*B*	*SE*	*B*	*SE*	*B*	*SE*
Perceived relationship with parents	0.523^***^	0.019	0.407^***^	0.028	0.436^***^	0.029
Perceived relationship with teachers	0.142^***^	0.018	0.259^***^	0.024	0.191^***^	0.031
Perceived relationship with friends	0.389^***^	0.016	0.346^***^	0.020	0.376^***^	0.025

***p < 0.001.

### 4.3 The direct and mediating role of sense of school belonging

The direct effect of students’ sense of school belonging on their well-being was examined using three regression models. First, for the full sample, the regression model had a good fit, with RMSEA (0.046) < 0.08, CFI (0.989) > 0.9, TLI (0.983) > 0.9, and SRMR (0.018) < 0.08. After controlling for other variables, students’ sense of school belonging explained 37.7% of variance in well-being, and the standardized regression coefficient was 0.614. Second, for the Grade 4 sample, the regression model had a good fit with RMSEA (0.036) < 0.08, CFI (0.992) > 0.9, TLI (0.988) > 0.9, and SRMR (0.013) < 0.08. After controlling for other variables, students’ sense of school belonging explained 36.5% of the variance in well-being, and the standardized regression coefficient was 0.604. Third, for the Grade 10 sample, the regression model had a good fit with RMSEA (0.060) < 0.08, CFI (0.982) > 0.9, TLI (0.974) > 0.9, and SRMR (0.029) < 0.08. After controlling for other variables, students’ sense of school belonging explained 31.8% of the variance in well-being, and the standardized regression coefficient was 0.564.

Three models were constructed to examine whether students’ sense of school belonging mediated the relationship between perceived parent–teacher–friend relationships and their well-being. The first model had a good fit, with RMSEA (0.038) < 0.08, CFI (0.981) > 0.9, TLI (0.976) > 0.9, and SRMR (0.034) < 0.08. The second model had a good fit, with RMSEA (0.030) < 0.08, CFI (0.986) > 0.9, TLI (0.983) > 0.9, and SRMR (0.021) < 0.08. The third model had a good fit, with RMSEA (0.045) < 0.08, CFI (0.972) > 0.9, TLI (0.966) > 0.9, and SRMR (0.040) < 0.08.

As illustrated in Figure 1 and Table 3, after controlling for other variables, students’ perceived parent–teacher–friend relationship and sense of school belonging explained 44.8% of variance in well-being for the full, 42.3% for Grade 4, and 36.9% for Grade 10 samples. It suggests that, compared to Grade 4 students, other variables can explain Grade 10 students’ well-being. Students’ perceived relationships with parents and friends not only had a direct effect on their well-being but also exhibited an indirect effect through the sense of school belonging for the full, Grade 4, and Grade 10 samples. However, Grade 4 students’ perceived relationship with teachers had a positive and significant effect on well-being, while their sense of school belonging also played a mediating role. For the full and Grade 10 samples, the direct effect of students’ perceived relationship with teachers on well-being was not significant, although the mediating role of their sense of school belonging was evident.

**Figure 1:**
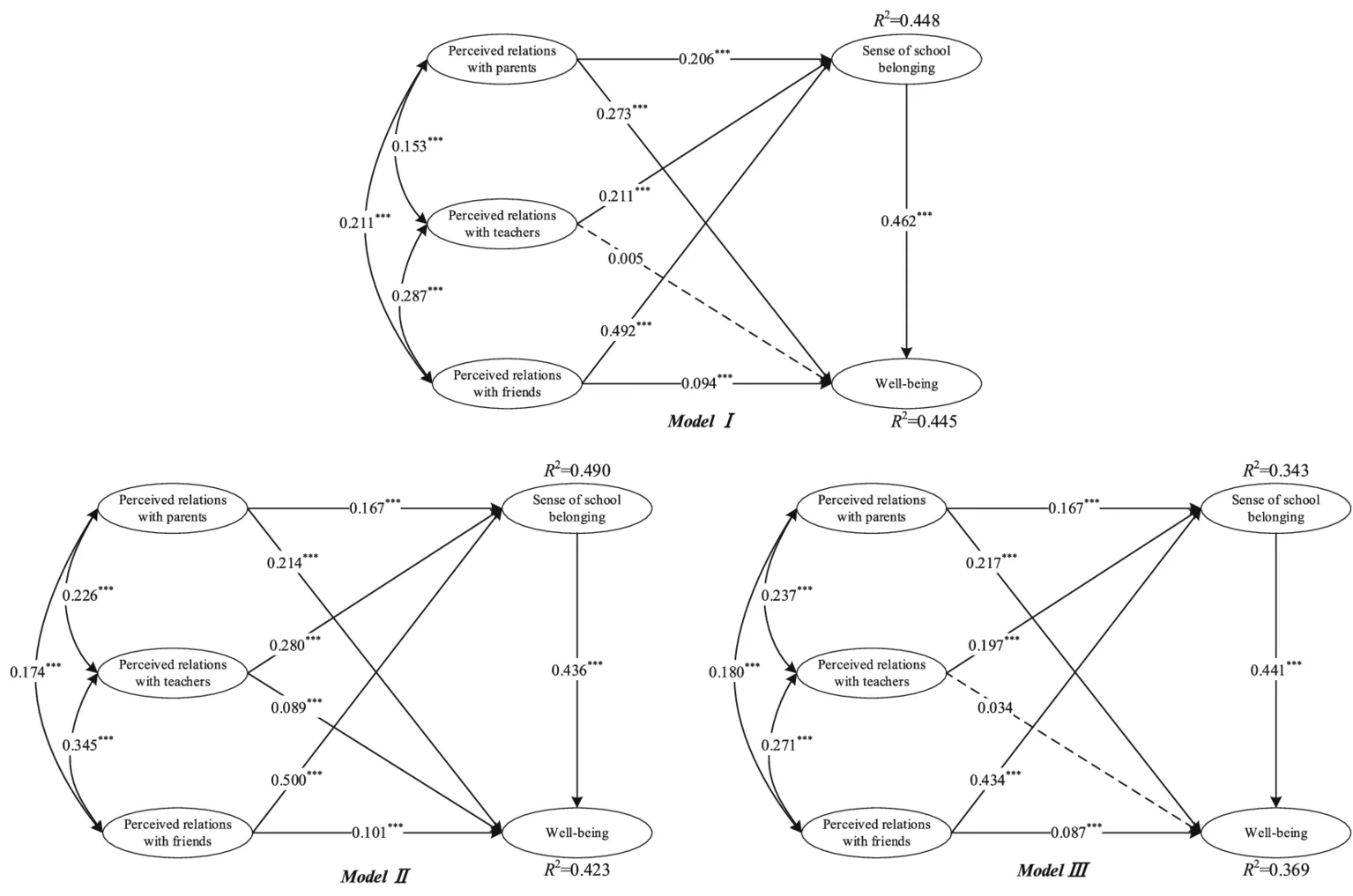
Structural equation modeling. Model I represents the full sample, Model II is the Grade 4 sample, and Model III reflects Grade 10 sample.

**Table 3:** Bootstrap analysis of significance test for intermediate effects.

Indirect path	Full sample (*n* = 6,241)			Grade 4 sample (*n* = 3,508)			Grade 10 sample (*n* = 3,234)		
	Estimate	95% CIs	*p*	Estimate	95% CIs	*p*	Estimate	95% CIs	*p*
Perceived relationship with parents → Sense of belonging at school → WHO-5 Index									
Total effect	0.368	0.339, 0.395	<0.001	0.277	0.234, 0.321	<0.001	0.291	0.253, 0.330	<0.001
Indirect effect	0.095	0.080, 0.114	<0.001	0.063	0.041, 0.086	<0.001	0.074	0.052, 0.100	<0.001
Direct effect	0.273	0.243, 0.302	<0.001	0.241	0.169, 0.258	<0.001	0.217	0.175, 0.260	<0.001
Perceived relationship with teachers → Sense of belonging at school → WHO-5 Index									
Total effect	0.103	0.075, 0.131	<0.001	0.211	0.170, 0.253	<0.001	0.121	0.077, 0.157	<0.001
Indirect effect	0.098	0.080, 0.116	<0.001	0.122	0.095, 0.152	<0.001	0.087	0.063, 0.113	<0.001
Direct effect	0.005	−0.024, 0.036	>0.05	0.089	0.042, 0.136	<0.001	0.034	−0.010, 0.068	>0.05
Perceived relationship with friends → Sense of belonging at school → WHO-5 Index									
Total effect	0.321	0.294, 0.348	<0.001	0.318	0.279, 0.359	<0.001	0.287	0.240, 0.316	<0.001
Indirect effect	0.227	0.202, 0.256	<0.001	0.218	0.182, 0.261	<0.001	0.192	0.159, 0.226	<0.001
Direct effect	0.094	0.058, 0.128	<0.001	0.101	0.051, 0.151	<0.001	0.087	0.035, 0.132	<0.001

## 5 Discussion

Drawing on data from SEES participants in Suzhou, China, this study explored the relationships among students’ perceived parent-teacher-student relationships, sense of school belonging, and well-being, while also examining differences between primary and secondary students. These findings establish the importance of parent-teacher-peer relationships for students’ sense of school belonging and well-being and identify the psychological mechanisms that explain both their effects and variation across developmental stages.

Based on the results of correlation analysis, regression analysis and the structural equation model, the quality of the relationship between parents, teachers and peers perceived by students is significantly positively correlated with their well-being. This shows that important interpersonal relationships in daily life may be associated with students’ psychological well-being to a certain extent. This finding is consistent with the general trend of international research in recent years. For example, parental support is an important predictor of adolescent well-being (Wu and Lee, 2022; Fino et al., 2025). The quality of the teacher-student relationship has also been proven to improve students’ well-being and inhibit the occurrence of externalized problem behavior to a certain extent (Cornelius-White, 2007; Jamal et al., 2013; OECD, 2017). In addition, the emotional support provided by peers is also an important correlate of adolescents’ well-being (Fino et al., 2025; Wang et al., 2025). In the Chinese context, the data of this study show that students reported the highest scores on the dimension of parental support, which can be understood from the perspective of traditional Chinese culture. Chinese parents generally have high educational expectations for their children and devote more energy to their children’s daily care and academic development. Students’ positive perception of teacher-student relationships may stem from their long time together at school, which creates favorable conditions for establishment of positive interpersonal connections. In China, the teaching profession often carries a strong sense of professional identity and responsibility. This helps to promote a closer relationship between teachers and students, which in turn has a positive impact on students’ well-being. Regarding the peer dimension, students tend to gain positive emotional experiences in interacting with peers who offer understanding, acceptance and respect.

The sense of school belonging acts not only as a direct predictor of well-being in the framework of this research, but also serves as a mediator between interpersonal relationships and well-being. The analysis results show that students’ perceived parent-teacher-student relationships, the sense of school belonging, and well-being form a significant positive correlational chain. Specifically, the quality of interpersonal relationships is positively associated with the sense of school belonging (Allen et al., 2018; Dukynaite and Dudaite, 2017), and the enhancement of the sense of belonging further promotes well-being. This enhancement, in turn, promotes positive identity formation during adolescence (Brechwald and Prinstein, 2011). The need-to-belong theory also supports this mediating path (Baumeister and Leary, 1995). Students experience frequent positive interactions in important relationships such as those with parents, teachers, and peers, from which they gain a sense of school belonging. Driven by the sense of school belonging, students are likely to experience well-being.

Regarding developmental stage differences, the differences between primary and secondary students revealed by this study manifest at multiple levels. First, primary school students have a positive perception of their relationships with parents and peers, while their evaluation of the teacher-student relationship is relatively negative, and the average sense of belonging and overall well-being of primary students are higher than those of secondary students. Second, after controlling for the relevant covariates, the structural equation model shows that the three types of interpersonal relationships perceived by primary students exhibit strong explanatory power for their sense of school belonging and well-being. Finally, under the same control conditions, the teacher-student relationship perceived by secondary students is not significantly associated with their well-being, while this path shows a significant positive effect in the group of primary students. In view of the above differences, this study attempts to interpret the educational context of primary and secondary school students in China. The need-to-belong theory points out that after individuals enter puberty, the threshold for satisfying the need to belong increases, and more frequent and high-quality social interactions are needed to achieve the same level of satisfaction. However, the substitutability of interpersonal relationships within the school tends to decline. Specifically, on the one hand, Chinese secondary students spend a long time at school every day, bearing a heavy academic burden and examination pressure, especially the fierce competition in the high school entrance examination. The relevant OECD report also points out that the level of exam anxiety is on the rise from childhood to adolescence (OECD, 2022). At the same time, teenagers are in a critical period of autonomous development. The psychological distance between them and their families is gradually increasing, and the importance of peer groups grows (Eccles, 1999). Against this background, with the increase in this satisfaction threshold, the independent predictive effect of the teacher-student relationship on secondary students’ well-being is not significant in this study, and this result is supported at the theoretical level. On the other hand, the transition from primary to secondary involves multiple adaptation tasks. At this stage, students may reduce their willingness to communicate with their parents and teachers, and rely more on their peers to gain support, recognition and a sense of school belonging. This aligns with Erikson’s concern that teenagers attach importance to peer recognition in the process of identity formation. Secondary teachers usually have high academic expectations of students. Compared with primary teachers, they may provide less emotional support, which also weakens the positive contribution of teacher-student relationships to well-being to a certain extent. This inference is consistent with the existing empirical findings: the level of school belonging of secondary students is generally lower than that of primary students (Morinaj and Hascher, 2022; Neel and Fuligni, 2012), and their level of well-being is also on the decline (Konu and Lintonen, 2006). In addition, several factors not included in this model may have a stronger association with the well-being of secondary students, including parental expectations, in-school and out-of-school homework load, exam anxiety and online leisure time (Arslan, 2021, 2022; OECD, 2017; Parr et al., 2020). In addition to the above stressful factors, the social network of secondary students is more extensive than that of primary students (Cai et al., 2023). Out-of-school friendships and online communities can compensate for the lack of school belonging to a certain extent (Cai et al., 2023). This phenomenon coexists with the aforementioned decline in the substitutability of intra-school relationships, which is also in line with the alternative satisfaction mechanism proposed by the belongingness hypothesis, and may further weaken the impact of school belonging on well-being.

Besides, it should be pointed out that the data of this study were only collected from Suzhou, which has a relatively developed economy in eastern China. The geographical limitation of the sample source means that the research conclusions need to be treated cautiously when promoted to areas with different socio-economic conditions or educational contexts. There may be different manifestations in the interpersonal interaction patterns and the functional positioning of school belonging in these areas.

## 6 Limitations

As this study utilized publicly available OECD data, the researchers were unable to determine the design of the variables and their corresponding measurement approaches, resulting in the three main predictor variables used in this study being measured in different ways. This makes it impossible to directly compare the effect sizes of the three relationship variables, as their different measurement metrics may affect the magnitude of the standardized coefficients. To enable meaningful effect size comparisons, future research should use the same measurement instruments for all three relationship dimensions. As noted above, students are situated within diverse school and family contexts, and factors such as school belonging and well-being are shaped by both individual student-level and organizational-level factors arising from the classroom, school, and family. Furthermore, the cross-sectional design of this study limits causal interpretation of the mediation pathways. Therefore, a multilevel linear model would be more appropriate for examining students’ well-being, Longitudinal or experimental designs are needed to establish the causal direction of the observed associations in future research. Furthermore, the validity coefficient for the students’ sense of school belonging scale was 0.674, which fell slightly below the commonly accepted threshold of 0.70. Future studies should consider using a well-validated scale with stronger psychometric properties in order to replicate these findings with greater measurement precision. Another limitation concerns the temporal gap between data collection and the current policy context. The SSES data used in this study were collected in Suzhou, China, in November 2019, yet the Chinese research team did not publish the findings until September 2021. More importantly, the implementation of China’s “Double Reduction” policy in 2021 has reshaped students’ school experiences. Students now spend more time on campus with increased opportunities to interact with teachers and peers, a factor that may have strengthened these relationships compared to the pre-policy period. Consequently, while our findings inform our understanding of the 2019 context, their generalizability to the post-2021 environment may be constrained. Future research using more recent datasets is needed to re-examine these relationships.

## 7 Implications

Students’ well-being serves as an important indicator that is not only positively associated with their current learning and development but may also be relevant to their future success. The study also found that while Chinese students are high achievers, they do not report a higher sense of school belonging, which remains lower than the OECD average (De Bortoli, 2018). Accordingly, it is imperative for school authorities, teachers, and parents to create a learning environment that promotes students’ aspirations and academic development. Parents, especially those whose children are in secondary school, should provide a warm, understanding, accommodating, and caring family environment, reduce excessive expectations of academic performance, and establish healthy parent–child relationships. Our finding that perceived teacher–student relationship quality among Grade 4 students is directly associated with well-being suggests that, in line with the need-to-belong theory, teachers may serve as a more direct relational source through which younger students’ need to belong is fulfilled. However, we also found that Grade 10 students’ perceived teacher–student relationship quality is associated with well-being only indirectly through school belonging. All teachers should be student-oriented, treat all students fairly, understand the challenges they face, and provide timely feedback. Secondary teachers should prioritize cultivating a broader sense of school community, rather than focusing on building rapport with individual students. This can be achieved by creating positive classroom environments, supporting peer mentoring programs, amplifying student voice, and acting as belonging facilitators. Meanwhile, schools and teachers should devote greater attention to disadvantaged students, especially those from lower socio-economic status families as they may have access to fewer resources and support systems than most of their peers.

## 8 Conclusion

The sense of school belonging plays a mediating role in the influence of students’ relationships with their parents, teachers, and peers on their well-being. According to the core viewpoint of ecological systems theory, the formation of school belonging is jointly influenced by multiple social-level factors, specifically including family interaction patterns, teacher-student relationships and peer-interaction relationships. The need-to-belong theory reveals the core value of school belonging for student development from the perspective of satisfying students’ fundamental need for social contentedness. The satisfaction of such basic needs builds a critical mechanism of action through which interpersonal-support resources obtained externally by individuals can be transformed into their own well-being experience.

These findings also reveal developmental differences across school levels. In practice, the mediating role of belonging identifies it as a clear intervention target. Families should provide warmth and support without excessive academic pressure. Schools should foster belonging, with primary teachers building close teacher–student rapport and secondary teachers should prioritize fostering a broader sense of school community over building rapport with individual students, by creating positive classroom environments, supporting peer mentoring, amplifying student voice, and acting as belonging facilitators. Several limitations warrant mention. The cross-sectional design limits causal inferences about the mediation pathways. Meanwhile, data were collected exclusively from Suzhou, an economically-developed city in eastern China. The generalizability of its findings is thus limited, and the results cannot be directly generalized to students from contexts with different socioeconomic development levels and diverse cultural backgrounds.

## Data Availability

The datasets for this study can be found on the official website of the Organization for Economic Cooperation and Development at: https://www.oecd.org/education/ceri/social-emotional-skills-study/data.htm. All researchers can download the database and related reports.
